# Effects of Sex and Estrous Cycle on the Time Course of Incubation of Cue-Induced Craving following Extended-Access Cocaine Self-Administration

**DOI:** 10.1523/ENEURO.0054-21.2021

**Published:** 2021-08-10

**Authors:** Claire M. Corbett, Emily Dunn, Jessica A. Loweth

**Affiliations:** 1 Graduate School of Biomedical Sciences, School of Osteopathic Medicine, Rowan University, Stratford, New Jersey 08084; 2 Department of Cell Biology and Neuroscience, Rowan University School of Osteopathic Medicine, Stratford, New Jersey 08084

**Keywords:** cocaine, drug seeking, estrous cycle, incubation, sex differences

## Abstract

Cocaine addiction is a devastating public health epidemic that continues to grow. Studies focused on identifying biological factors influencing cocaine craving and relapse vulnerability are necessary to promote abstinence in recovering drug users. Sex and ovarian hormones are known to influence cocaine addiction liability and relapse vulnerability in both humans and rodents. Previous studies have investigated sex differences in the time-dependent intensification or “incubation” of cue-induced cocaine craving that occurs during withdrawal from extended-access cocaine self-administration and have identified changes across the rat reproductive cycle (estrous cycle). Female rats in the estrus stage of the cycle (Estrus Females), the phase during which ovulation occurs, show an increase in the magnitude of incubated cue-induced cocaine craving compared with females in all other phases of the estrous cycle (Non-Estrus Females). Here we extend these findings by assessing incubated craving across the estrous cycle during earlier withdrawal periods (withdrawal day 1 and 15) and later withdrawal periods (withdrawal day 48). We found that this increase in the magnitude of incubated craving during estrus (Estrus Females) is present on withdrawal day 15, but not on withdrawal day 1, and further increases by withdrawal day 48. No difference in the magnitude of incubated craving was observed between Males and Non-Estrus Females. Our data indicate that the effects of hormonal fluctuations on cue-induced cocaine craving intensify during the first month and a half of withdrawal, showing an interaction among abstinence length, estrous cycle fluctuations, and cocaine craving.

## Significance Statement

Cocaine addiction is a chronic, relapsing condition. Females have been historically understudied in preclinical research studies but understanding how biological factors like sex and ovarian hormones influence relapse vulnerability is critical in our understanding of how to reduce craving and promote abstinence in recovering users of both sexes. Here we characterized changes in the intensification or “incubation” of cue-induced cocaine craving during earlier and later withdrawal from extended-access cocaine self-administration between male and female rats and across the estrous cycle. Our results indicate an interaction between estrous cycle fluctuations and time-dependent changes in the underlying craving state and lay the foundation for future studies focused on investigating cellular mechanisms driving sex differences in relapse vulnerability.

## Introduction

Cocaine addiction is a chronic, relapsing disorder that continues to pose a serious public health problem. Alarmingly, deaths from cocaine overdose in the United States doubled from 2011 to 2016 (Kampman, 2019), and relapse rates have remained high for decades (Hunt et al., 1971; Sinha, 2011). Sex is known to influence cocaine addiction liability and relapse vulnerability, with women becoming addicted to cocaine more rapidly than men and showing more serious drug-related health complications, effects which are influenced by fluctuations in ovarian hormones across the menstrual cycle (Greenfield et al., 2010; Carroll and Smethells, 2016). In rodents, motivation to obtain and seek for cocaine is also influenced by fluctuations of ovarian hormones across the rat reproductive cycle (estrous cycle; Carroll and Anker, 2010; Carroll and Lynch, 2016). However, less is known regarding how abstinence length and estrous cycle fluctuations interact to influence cocaine craving and relapse vulnerability following extended-access cocaine self-administration.

Drug-associated cues are known to elicit craving and are one of the most common relapse triggers. Cue-induced cocaine craving has been shown to increase or “incubate” over time in both humans (Parvaz et al., 2016) and rats (Grimm et al., 2001; Lu et al., 2004; Loweth et al., 2014a,b; Zlebnik and Carroll, 2015; Glynn et al., 2018; Nicolas et al., 2019), a phenomenon referred to as incubation of cue-induced craving. This time-dependent increase in cue-induced craving occurring during forced abstinence or withdrawal is thought to lead to increased relapse vulnerability. Recent preclinical studies in intact, freely cycling rodents following extended-access cocaine self-administration (8 h) have shown changes in incubated craving across the estrous cycle (Nicolas et al., 2019), which lasts 4–5 d and consists of four major phases (metestrus, diestrus, proestrus, and estrus) over which estrogen and progesterone levels fluctuate (Becker et al., 2005; Lebron-Milad and Milad, 2012; see Materials and Methods). Specifically, while no differences in seeking behavior are observed across the estrous cycle on withdrawal day 2, an increase in the magnitude of incubated cue-induced craving is observed during estrus (“Estrus Females”) compared with females in all other stages of the estrous cycle (“Non-Estrus Females”) following 1 month of withdrawal (withdrawal day 29; Nicolas et al., 2019). Estrus is the cycle stage in which ovulation occurs and when estradiol levels have just dropped from peak levels, but the ratio of estradiol to progesterone remains slightly elevated (Broestl et al., 2018; Carney, 2019). These findings are in general agreement with others showing that both drug seeking under extinction conditions and cocaine-primed reinstatement of previously extinguished drug-seeking behavior is highest in Estrus Females compared with Non-Estrus Females and Males following short-access (2 h) cocaine self-administration (Kippin et al., 2005; Feltenstein and See, 2007; Kerstetter et al., 2008). Here we both replicate and extend previously reported findings (Nicolas et al., 2019) by assessing time-dependent changes in the magnitude of cue-induced cocaine craving in males and females and across the estrous cycle during withdrawal from extended-access cocaine self-administration.

## Materials and Methods

### Subjects and surgery

Male (weight on arrival, 250–275 g) and female (weight on arrival, 225–250 g) adult Sprague Dawley rats were purchased from Envigo. Rats were housed on a reverse light/dark cycle with food and water freely available (lights off at 9:00 A.M., lights on at 9:00 P.M.). All rats were given an initial acclimation period (5–7 d) during which rats were group housed by sex (two to three rats/cage). Rats were then anesthetized with ketamine and xylazine (males: 80 and 10 mg/kg, i.p.; females: 60 and 7.5 mg/kg, i.p.), and a SILASTIC catheter (Plastics One) was inserted into the right jugular vein and passed subcutaneously to the mid-scapular region. Rats were singly housed immediately after surgery and for the remainder of the study. All procedures were approved by the Institutional Animal Care and Use Committee and conducted in accordance with the US Public Health Service Guide for the Care and Use of Laboratory Animals.

### Cocaine self-administration

Following implantation of intravenous jugular catheters and 5–7 d of recovery, all rats underwent cocaine self-administration 6 h/d for 10 d under a fixed-ratio-1 (FR1) reinforcement schedule (0.5 mg/kg/infusion). Self-administration sessions started shortly after the onset of the dark cycle (∼10:00 A.M.) and were conducted in operant chambers (MED Associates) equipped with active and inactive nose-poke holes. Active hole responses turned on the infusion pump and led to the delivery of a 20 s light cue and a 20 s time-out period, while poking in the inactive hole was without consequence. Pump times for each rat/chamber were adjusted based on body weight (so as to deliver 0.5 mg/kg/infusion in a 100 μl/kg volume to all rats). During the time-out period, nose pokes in the active hole were recorded but did not result in an infusion. Twelve rats that did not learn to self-administer cocaine and/or had faulty catheters were excluded from the study and killed.

### Tests for cue-induced cocaine seeking

Seeking tests were conducted using a between-subjects design in which rats received a test on withdrawal day 1, 15, or 48 (30 min each). For the withdrawal day 15 and 48 time points, rats were tested within 1 d of the specific time point (e.g., 14–16, 47–49) to ensure that enough animals were in Estrus and Non-Estrus at the time of the test. Males were tested on an identical schedule to have an even distribution across withdrawal days within all groups. As with self-administration sessions, each seeking test began during the dark cycle (∼10:00 A.M.). During the seeking test, nose pokes in the active hole resulted in presentation of the light cue previously paired with cocaine, but no infusion. Responding in the inactive hole had no consequence and controls for general activity level. The number of times an animal responds in the active hole under these conditions is the operational measure of cue-induced cocaine craving (Grimm et al., 2001; Lu et al., 2004).

### Estrous cycle monitoring

Estrous cycle was determined in freely cycling females across self-administration training, throughout withdrawal, and before seeking tests. Vaginal swabs were taken regularly from all female rats throughout the study (4–5 d in a row followed by 2–3 d off) to effectively track the cycle of each animal. Females were swabbed at the onset of the dark cycle (∼9:00 to 10 A.M.) and ∼30 min before the start of any behavioral test to ensure that we knew the stage the rat was in when each session began. As described previously by other laboratories (Bangasser and Shors, 2008), vaginal samples were collected by gently swabbing the vaginal canal using a saline-dipped, cotton-tipped applicator and samples were “smeared” on glass microscope slides. Males were handled on an identical schedule. Slides were stained with toluidine blue and examined using light microscopy. The estrous cycle stage was determined by the presence and morphology of cells based on previously published criteria by other laboratories (Hubscher et al., 2005; Cora et al., 2015). Each smear was classified as one of the following four stages: metestrus (also known as diestrus I or D1); diestrus (also known as diestrus II or D2); proestrus; and estrus. The ovarian hormones estradiol and progesterone fluctuate across these four stages of the estrous cycle as follows: estradiol and progesterone levels are lower during metestrus and diestrus, and surge during proestrus (with the estradiol surge preceding the progesterone surge); both hormone levels decline during estrus, when ovulation occurs (Becker et al., 2005; Becker and Hu, 2008; Lebron-Milad and Milad, 2012). The ratio of estradiol to progesterone is lower during metestrus and diestrus, and elevated in proestrus and, to a lesser extent, in estrus (Broestl et al., 2018; Carney, 2019). Metestrus was classified based on the presence of approximately equal amounts of nucleated epithelial cells, non-nucleated cornified epithelial cells, and leukocytes. Diestrus was classified based on the observation of a few cells, including leukocytes and occasionally epithelial cells. The proestrus phase was classified based on the presence of ≥75% of nucleated epithelial cells, and the estrus phase (vaginal estrus) was classified by the presence of ≥75% of non-nucleated cornified epithelial cells. Although there have been some reports in the literature of cocaine exposure disrupting estrous cyclicity in rats in a dose-dependent manner (King et al., 1993), all female rats in this study were lavaged regularly (4–5 d in a row each week) throughout the entire course of the study and exhibited normal cycling (4–5 d cycles) through all four stages of the estrous cycle across the entire study.

### Data analysis

Behavioral data averaged across all 10 self-administration days or across an entire 30 min seeking test session were analyzed using a one-way or two-way ANOVA. Behavioral data analyzed across multiple days or time bins within a session were analyzed using a repeated-measures ANOVA and, in isolated cases where data were missing for a certain time point because of a computer malfunction or other technical issues, mean replacements were used. When a significant effect was observed, Tukey’s *post hoc* analyses were conducted. Data analyses were performed using Statistica (Tibco) and GraphPad Prism. Statistical significance was set at *p < *0.05. All data are expressed as the mean ± SEM.

## Results

### No effects of sex or estrous cycle on cocaine intake during extended-access cocaine self-administration

**Figure 1.**
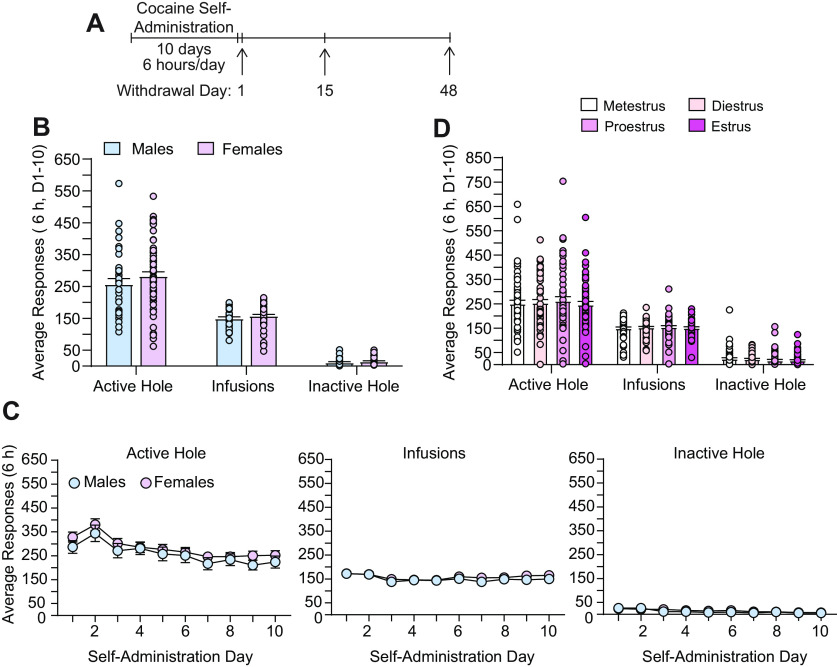
No effects of sex or estrous cycle on cocaine self-administration. ***A***, Experimental timeline. ***B***, No sex differences were observed for average active hole responding, infusions obtained, or inactive hole responding across the 10 d of cocaine self-administration. ***C***, No sex differences were observed in daily active hole responding, infusions obtained, or inactive hole responding over the 10 d self-administration period. ***D***, No estrous cycle effects were observed for average active hole responding, infusions obtained, or inactive hole responding across the 10 d of cocaine self-administration. Data are shown as the mean ± SEM (*n* = 36 males; *n* = 60 females).

Rats self-administered cocaine under extended-access conditions (6 h/d for 10 d, 0.5 mg/kg/infusion), and this was followed by forced abstinence. Incubation was assessed by administering cue-induced seeking tests during earlier (withdrawal day 1 or 15) or later (withdrawal day 48) time points (Fig. 1*A*, timeline). This self-administration regimen is known to produce incubation of cue-induced cocaine craving in both males (Lu et al., 2004; Loweth et al., 2014a; Glynn et al., 2018; Nicolas et al., 2019) and females (Zlebnik and Carroll, 2015; Nicolas et al., 2019). Consistent with previous reports from other laboratories in which animals self-administered cocaine under extended-access conditions (Nicolas et al., 2019), no difference in averaged responding (active or inactive hole nose pokes) or cocaine intake (infusions) was observed between males (*n* = 36) and females (*n* = 60) over the 10 d of self-administration (Fig. 1*B*). One-way ANOVAs conducted on these data averaged across all 10 self-administration days revealed no significant effect of sex (male, female) on the average number of active hole responses (*F*_(1,94)_ = 1.46, *p* = 0.23), infusions obtained (*F*_(1,94)_ = 1.38, *p* = 0.24), and inactive hole responses (*F*_(1,94)_ = 1.61, *p* = 0.21; Fig. 1*B*). Additional analyses of daily responding and cocaine intake across the 10 d self-administration period with sex as the between-subjects factor (male, female) and self-administration day as the within-subjects factor (days 1–10) similarly revealed no sex differences in cocaine self-administration (Fig. 1*C*). No significant effect of sex or interaction between sex and self-administration day on the number of active hole responses (*F*_(1,94)_ = 1.46, *p* = 0.23; *F*_(9,846)_ = 0.20, *p* = 0.99), infusions obtained (*F*_(1,94)_ = 1.38, *p* = 0.24; *F*_(9,846)_ = 0.58, *p* = 0.82), or inactive hole responses (*F*_(1,94)_ = 1.61, *p* = 0.21; *F*_(9,846)_ = 1.60, *p* = 0.11) was observed, although a significant effect of self-administration day was observed for all three measures (*F*_(9,846)_ = 9.05, *p* < 0.0001; *F*_(9,846)_ = 5.04, *p* < 0.0001; *F*_(9,846)_ = 14.46, *p* < 0.0001) because of slight variations in responding across all groups over the 10 d self-administration period (Fig. 1*C*).

To assess whether there was an effect of estrous cycle on responding or cocaine intake during self-administration, rats were lavaged daily to determine cycle stage on each of the 10 d of cocaine self-administration. Self-administration data were then analyzed across all four stages of the estrous cycle for each rat: metestrus, diestrus, proestrus, and estrus. Importantly, no effects of cocaine exposure on estrous cycling were observed, and rats followed a regular 4–5 d cycle throughout the study, during which they moved through each of the four cycle stages (metestrus, diestrus, proestrus, and estrus). Of the 60 females included in this study, two were excluded from this analysis because a clear proestrus smear (≥75% nucleated epithelial cells; see Materials and Methods) was not obtained at the time vaginal smears were taken. No differences in averaged responding (active or inactive hole nose pokes) or cocaine intake across the 10 d of self-administration were observed across the four stages of the estrous cycle (Fig. 1*D*). The one-way repeated-measures ANOVA conducted on these data (*n* = 58 females) with cycle as the within-subjects factor revealed no significant effect of estrous cycle (metestrus, diestrus, proestrus, estrus) on the average number of active hole responses (*F*_(3,171)_ = 0.44, *p* = 0.73), infusions obtained (*F*_(3,171)_ = 0.54, *p* = 0.66), and inactive hole responses (*F*_(3,171)_ = 1.34, *p* = 0.26; Fig. 1*D*). Together, these findings show that there were no sex differences or estrous cycle effects on active and inactive hole responding or cocaine intake during extended-access cocaine self-administration (Fig. 1).

### Time-dependent effects of estrous cycle fluctuations on incubation of cocaine craving

**Figure 2.**
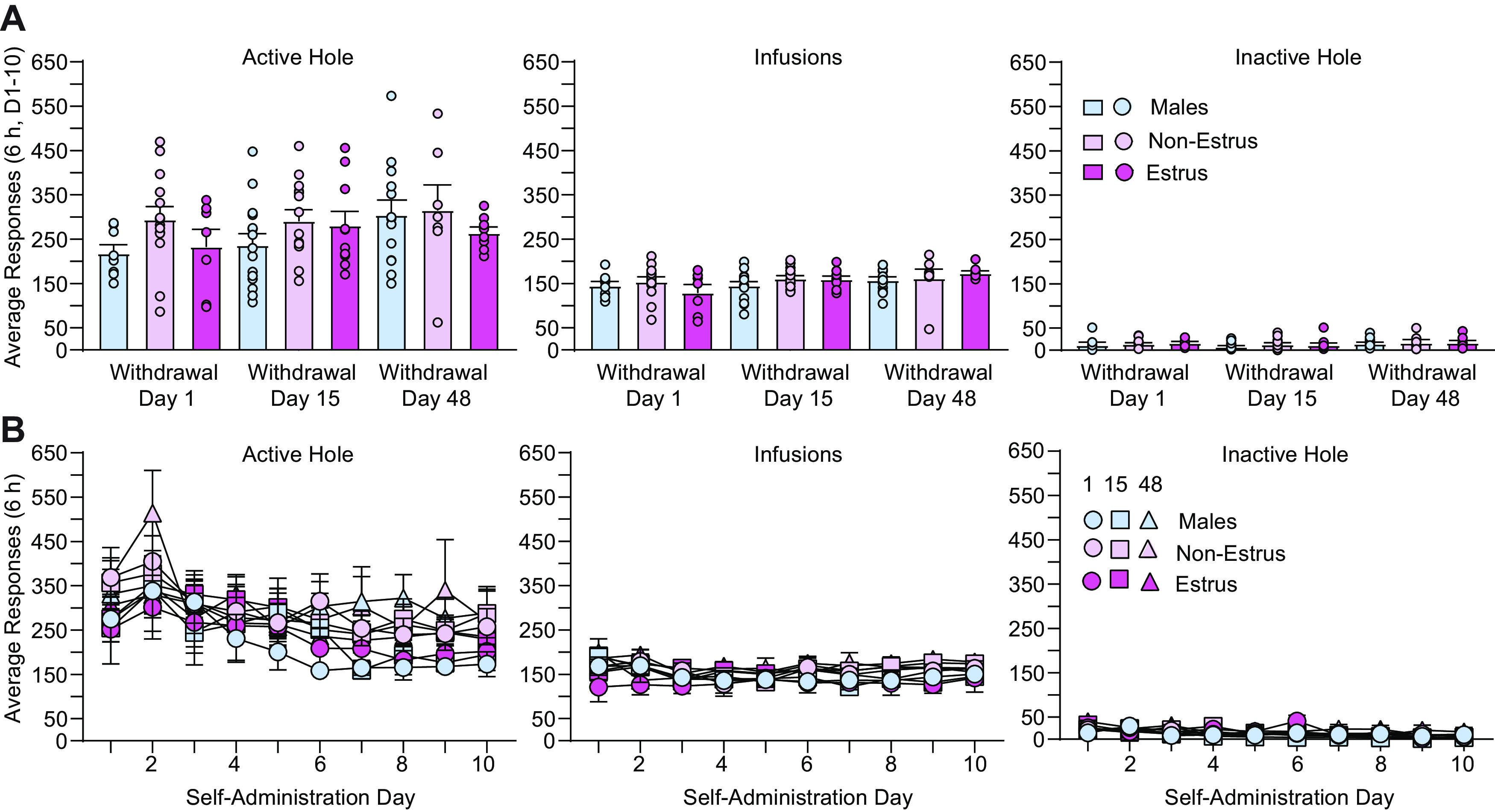
No differences in cocaine self-administration across groups tested at different withdrawal time points. ***A***, ***B***, No group differences (for animals destined for the nine groups shown in Fig. 3) were observed in average (days 1–10, ***A***) or daily (***B***) active hole responding, infusions obtained, or inactive hole responding (as described in Fig. 1) during cocaine self-administration, indicating that group differences in incubated cocaine-seeking behavior (Fig. 3) were not because of differences in cocaine intake or self-administration behavior.

**Figure 3.**
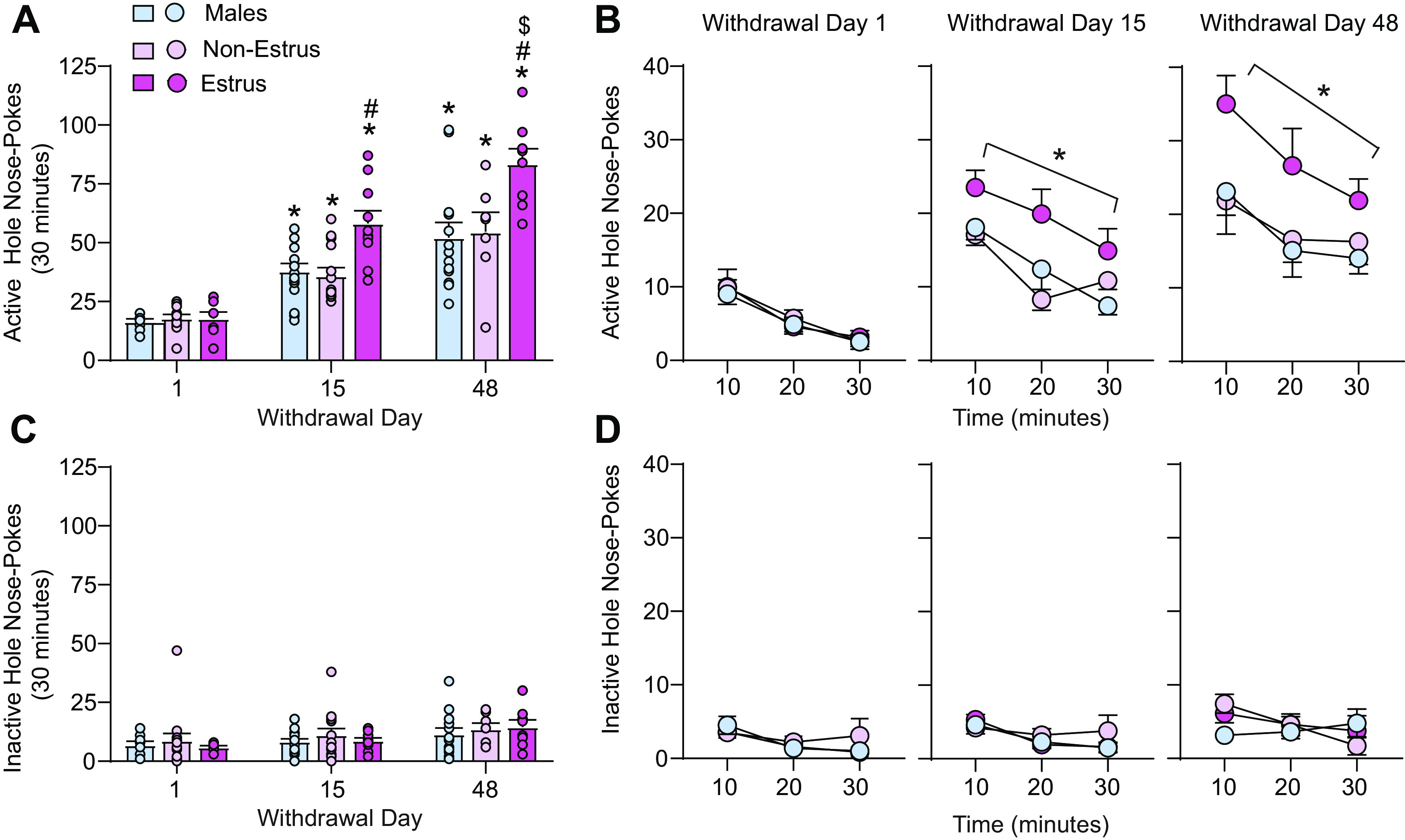
Estrus Females show a time-dependent increase in the magnitude of incubated cue-induced cocaine craving compared with both Non-Estrus Females and Males. ***A***, Active hole responding on withdrawal days 1, 15, and 48. Data are shown as the mean ± SEM number of active hole (paired with the light cue) nose pokes during each seeking test (30 min). While all three groups demonstrated incubation of cue-induced cocaine craving over the first month and a half of withdrawal, Estrus Females showed an increase in the magnitude of incubated cue-induced craving compared with both Non-Estrus Females and Males that was present on withdrawal day 15 and continued to increase on withdrawal day 48. *p < 0.05, versus corresponding withdrawal day 1 for each group; #*p* < 0.05, versus Non-Estrus Females and Males on the specified time point; $*p* < 0.05, versus Estrus on withdrawal day 15. ***B***, Active hole responding (mean ± SEM) across each 30 min seeking test in 10 min time bins. *p < 0.05, versus Non-Estrus Females and Males averaged across all time bins. ***C***, Inactive hole responding on withdrawal day 1, 15, and 48. Data are shown as the mean ± SEM number of inactive hole (without consequence) nose pokes during each seeking test (30 min). *p < 0.05, withdrawal day 48 versus withdrawal day 1 averaged across all groups. ***D***, Inactive hole responding (mean ± SEM) across each 30 min seeking test. *n* = 7–15 rats/group.

To determine the time course of known sex differences in incubation of cue-induced craving across the estrous cycle, separate groups of rats received cue-induced seeking tests during earlier (withdrawal days 1 or 15) and later (withdrawal day 48) withdrawal time points (Fig. 1*A*, timeline), and no group differences in cocaine self-administration behavior were observed across groups assigned to different withdrawal time points (Fig. 2; see description of analyses below). On withdrawal days 15 and 48, all rats were tested within 1 d of the seeking test time point to ensure an adequate number per group across the estrous cycle (see Materials and Methods). These withdrawal periods were chosen based on previously published reports in male rats showing that craving is elevated but not maximal during the first few weeks of withdrawal (e.g., withdrawal day 15) and incubates further over the next month during withdrawal from extended-access cocaine self-administration (Lu et al., 2004; Loweth et al., 2014b; Glynn et al., 2018). During the cue-induced seeking test, responding in the previously active hole delivered only the cue (no cocaine) while responding in the inactive hole was without consequence; active hole nose pokes under this condition provide an operational measure of cue-induced drug seeking. During withdrawal, rats were lavaged daily for at least 4–5 consecutive days to determine estrous cycle stage. To control for this, males were handled on an identical schedule. As expected based on previous reports (Kerstetter et al., 2008; Nicolas et al., 2019), analyses revealed no difference in cue-induced seeking behavior between females in metestrus, diestrus, and proestrus (commonly referred to as Non-Estrus Females) on each withdrawal day. One-way ANOVAs conducted on active hole nose pokes across each of these three cycle stages at each withdrawal time revealed no significant group differences, as follows: withdrawal day 1 (*n* = 14), *F*_(2,11)_ = 0.84, *p* = 0.46; withdrawal day 15 (*n* = 14), *F*_(2,11)_ = 1.13, *p* = 0.36; and withdrawal day 48 (*n* = 7), *F*_(2,4)_ = 0.26, *p* = 0.78. Thus, all rats in these three stages on each withdrawal day (1, 15, and 48) were combined into a Non-Estrus Females group and compared with rats in estrus (Estrus Females) and Males (Fig. 3), as others have done previously (Kippin et al., 2005; Kerstetter et al., 2008; Nicolas et al., 2019). To ensure that group differences in incubation of cue-induced seeking behavior (Fig. 3) could not be attributed to group differences in cocaine intake or responding during self-administration, self-administration behavior was analyzed across all nine groups (Fig. 2). One-way ANOVAs conducted on these data revealed no significant differences in average active hole responding (*F*_(8,87)_ = 1.16, *p* = 0.36), inactive hole responding (*F*_(8,87)_ = 0.69, *p* = 0.69), or infusions obtained (*F*_(8,87)_ = 1.23, *p* = 0.34) across the 10 d of self-administration (Fig. 2*A*). Additional analyses of daily responding and cocaine intake across the 10 d self-administration period with treatment group as the between-subjects factor (nine groups) and self-administration day as the within-subjects factor (days 1 through 10) similarly revealed no group differences in cocaine self-administration (Fig. 2*B*). No significant group effect or interaction between treatment group and self-administration day on the number of active hole responses (*F*_(8,87)_ = 1.16, *p* = 0.34; *F*_(72,783)_ = 0.57, *p* = 0.99) or infusions obtained (*F*_(8,87)_ = 1.29, *p* = 0.26; *F*_(72,783)_ = 0.82, *p* = 0.86) was observed. No significant group effect was observed for the number of inactive hole responses (*F*_(72,783)_ = 0.69, *p* = 0.69), although a small interaction between treatment group and self-administration day was observed (*F*_(72,783)_ = 1.41, *p* = 0.02; Fig. 2*B*). As in Figure 1, a significant effect of self-administration day was observed for all three measures (active hole, infusions, inactive hole: *F*_(9,783)_ = 9.11, *p* < 0.0001; *F*_(9,783)_ = 3.58, *p* = 0.0002; *F*_(9,783)_ = 11.86, *p* < 0.0001, respectively) because of slight variations in responding across all groups over the 10 d self-administration period (Fig. 2*B*).

Analyses of seeking test data (Fig. 3*A*) revealed that although all three groups (Males, Non-Estrus, Estrus) exhibited similar active hole nose pokes during the seeking test on withdrawal day 1 and a progressive increase in seeking behavior on withdrawal day 15 and 48 (i.e., incubation), the magnitude of the seeking responses on withdrawal days 15 and 48 was significantly greater in Estrus Females compared with both Non-Estrus Females and Males. Thus, the two-way ANOVA conducted on these data with sex/cycle (Males, Non-Estrus, Estrus) and withdrawal day (1, 15, and 48) as the between-subjects factors revealed a significant effect of sex/cycle (*F*_(2,87)_ = 11.98, *p* < 0.0001) and withdrawal (*F*_(2,87)_ = 63.85, *p* < 0.0001), and a significant interaction between sex/cycle and withdrawal (*F*_(4,87)_ = 2.99, *p* = 0.023). Tukey’s *post hoc* tests revealed a significant increase in cue-induced seeking on withdrawal day 15 and 48 compared with seeking on withdrawal day 1 (*p < 0.05) in each group and a significant increase in cue-induced seeking in Estrus Females compared with both Males and Non-Estrus Females on withdrawal days 15 and 48 (Fig. 3*A*, #*p* < 0.05). Notably, the magnitude of incubated craving in Estrus Females was higher on withdrawal day 48 than withdrawal day 15 (Fig. 3*A*, $*p* < 0.05), indicating that they did not simply reach a maximal level of responding more quickly than Non-Estrus Females and Males. Similar to total responding (Fig. 3*A*), analyses of active hole nose pokes across the 30 min session on each withdrawal time point revealed enhanced drug seeking in Estrus Females compared with both Males and Non-Estrus Females on withdrawal days 15 and 48 (Fig. 3*B*). The between-subjects/within-subjects ANOVAs conducted at each time point with sex/cycle (Males, Non-Estrus, Estrus) as the between-subjects factor and time (10, 20, and 30 min) as the within-subjects factor revealed a significant effect of time on each withdrawal test day, since, as expected, drug seeking was highest during the first 10 min and gradually decreased across each session (withdrawal day 1: *F*_(2,52)_ = 22.8, *p* < 0.0001; withdrawal day 15: *F*_(2,72)_ = 14.59, *p* < 0.0001; withdrawal day 48: *F*_(2,50)_ = 6.37, *p* = 0.0034). While no significant effect of sex/cycle (*F*_(2,26)_ = 0.26, *p* = 0.77) was observed on withdrawal day 1, a significant main effect of sex/cycle was observed on withdrawal days 15 (*F*_(2,36)_ = 8.90, *p* = 0.0007) and 48 (*F*_(2,25)_ = 5.74, *p* = 0.0089), while no significant interaction was observed at any time point (withdrawal day 1: *F*_(4,52)_ = 0.15, *p* = 0.96; withdrawal day 15: *F*_(4,72)_ = 1.39, *p* = 0.25; withdrawal day 48: *F*_(4,50)_ = 0.3015, *p* = 0.88). Tukey’s *post hoc* tests conducted on the main effect of sex/cycle revealed an overall significant increase in active hole responding in Estrus Females compared with both Males and Non-Estrus Females on withdrawal days 15 (*p* = 0.002, *p* = 0.001, respectively) and 48 (*p* = 0.01, *p* = 0.04, respectively; Fig. 3*B*). Importantly, no effect of sex or estrous cycle on inactive hole responding was observed (Fig. 3*C*), indicating that estrous cycle fluctuations selectively influence drug-seeking behavior. The two-way ANOVA conducted on inactive hole responding with sex/cycle (Males, Non-Estrus, Estrus) and withdrawal day (1, 15, and 48) as the between-subjects factors revealed no significant effect of sex/cycle (*F*_(2,87)_ = 0.73, *p* = 0.48) or interaction between sex/cycle and withdrawal (*F*_(4,87)_ = 0.14, *p* = 0.96). A small but significant effect of withdrawal (*F*_(2,87)_ = 3.84, *p* = 0.03) was observed in all groups because of a very small increase in inactive hole responding on withdrawal day 48 compared with withdrawal day 1 (*p* = 0.03). The between-subjects/within-subjects ANOVAs conducted on inactive hole responses at each time point with sex/cycle (Males, Non-Estrus, Estrus) as the between-subjects factor and time (10, 20, and 30 min) as the within-subjects factor similarly revealed no significant effect of sex/cycle (withdrawal day 1: *F*_(2,26)_ = 0.28, *p* = 0.75; withdrawal day 15: *F*_(2,36)_ = 0.69, *p* = 0.51; withdrawal day 48: *F*_(2,25)_ = 0.34, *p* = 0.71), and no significant interaction between sex/cycle and time (withdrawal day 1: *F*_(4,52)_ = 0.51, *p* = 0.73; withdrawal day 15: *F*_(4,72)_ = 0.79, *p* = 0.53; withdrawal day 48: *F*_(4,50)_ = 1.91, *p* = 0.12), although slight variations in inactive hole responding were observed across each session (withdrawal day 1: *F*_(2,52)_ = 2.93, *p* = 0.06; withdrawal day 15: *F*_(2,72)_ = 5.13, *p* = 0.01; withdrawal day 48: *F*_(2,50)_ = 1.84, *p* = 0.17; Fig. 3*D*). These studies are the first to investigate the time course of incubated cue-induced craving across the estrous cycle in females following extended-access self-administration and show an increase in the magnitude of craving in Estrus Females compared with Non-Estrus Females and Males as withdrawal progresses (i.e., more robust incubation of craving; Fig. 3*A*).

## Discussion

This study investigated the effects of sex and estrous cycle on the time course of incubation of cue-induced cocaine craving in adult male and female rats during withdrawal from extended-access cocaine self-administration. While no sex differences in cocaine self-administration were observed, we found an increase in the magnitude of incubated cue-induced craving in Estrus Females compared with both Non-Estrus Females and Males that was present on withdrawal day 15 and was further elevated on withdrawal day 48 (Fig. 3). Our data indicate that the effects of hormonal fluctuations on cue-induced cocaine craving and relapse-like behavior are influenced by abstinence length, findings which have important implications for treatment strategies focused on promoting abstinence in recovering users of both sexes.

### Effects of sex and ovarian hormones on cocaine addiction and relapse vulnerability

While our findings are the first to investigate the time course of incubated craving across the estrous cycle during withdrawal from extended-access cocaine self-administration, ovarian hormones are known to significantly influence cocaine addiction liability and relapse vulnerability. During the follicular phase of the menstrual cycle when estrogen levels rise and progesterone levels are low, females report a greater high from cocaine administration (Evans et al., 2002; Evans and Foltin, 2010). During the luteal phase, when estrogen levels are declining and progesterone levels are increasing, women have reported reduced cardiovascular and subjective effects to cocaine (Evans and Foltin, 2010). Similarly, progesterone levels are inversely related to cocaine craving as women with higher progesterone levels show lower cue-induced cocaine craving compared with women with lower progesterone levels (Sinha et al., 2007).

Preclinical studies in freely cycling rodents have also shown that regulation of cocaine intake, motivation to obtain cocaine, and cue- and cocaine-primed reinstatement of drug-seeking behavior change across the rat reproductive (estrous) cycle. These behaviors are enhanced during estrus (Estrus Females), when estrogen levels are declining but the ratio of estradiol to progesterone remains slightly elevated (Carney, 2019), compared with females in all other cycle stages (Roberts et al., 1989; Lynch et al., 2000; Kippin et al., 2005; Feltenstein and See, 2007). In addition, time-course studies conducted following short-access (2 h) cocaine self-administration found enhanced drug seeking both under extinction conditions and following cocaine-primed reinstatement in Estrus Females compared with Non-Estrus Females and Males up to 6 months after the last self-administration session (Kerstetter et al., 2008). Our data and recent findings from Nicolas et al. (2019) conducted following extended-access cocaine self-administration regimens are generally consistent with these previous findings and indicate an increase in the magnitude of incubated cue-induced cocaine craving in Estrus Females compared with Non-Estrus Females and Males on both withdrawal days 15 and 48 (Fig. 3; withdrawal day 29; Nicolas et al., 2019). In both studies conducted during withdrawal from extended-access cocaine self-administration, no effect of estrous cycle was observed on cue-induced cocaine seeking during the first day or two of withdrawal (withdrawal day 1, Fig. 3; withdrawal day 2, Nicolas et al., 2019). Although seeking is low at this time point, studies have shown that group differences in cue-induced cocaine-seeking behavior can be detected on withdrawal day 1 from extended-access cocaine self-administration following attenuation of BDNF–TrkB signaling in the nucleus accumbens (NAc; Li et al., 2013). Significant increases in responding under extinction conditions following short-access cocaine self-administration have also been observed in females compared with males (Fuchs et al., 2005; Kippin et al., 2005) and across the estrous cycle on withdrawal day 1, with Estrus Females showing enhanced responding on the active lever (previously paired with cocaine and a discrete cue) compared with Non-Estrus Females and Males (Feltenstein and See, 2007; Kerstetter et al., 2008; see below). Such findings argue against a floor effect and instead suggest that the effects of estrous cycle fluctuations on the incubation of craving during withdrawal from extended-access cocaine self-administration may be influenced by the underlying craving state, which progressively increases or incubates during the first month of withdrawal. Future studies are needed to determine when estrous cycle-dependent changes in incubated craving develop between withdrawal days 1 and 15 and to better understand sex differences in drug seeking on withdrawal day 1 across short-access models (Kippin et al., 2005; Fuchs et al., 2005; Feltenstein and See, 2007) and extended-access models (Nicolas et al., 2019; Fig. 3), as lower extinction responding has been observed on withdrawal day 1 following extended-access compared with short-access regimens (Mantsch et al., 2004; Sorge and Stewart, 2005). Although not observed in the current study (see Materials and Methods), the potential impact of cocaine-induced disruptions in estrous cyclicity (King et al., 1993) on withdrawal-dependent changes in drug-seeking behavior across different cocaine self-administration models must also be addressed.

While hormonal suppression and replacement studies have not been conducted with the incubation model, suppressing ovarian hormones via ovariectomy has been shown to reduce cocaine-primed reinstatement of drug-seeking behavior while chronic estradiol replacement in ovariectomized animals restores drug seeking to levels observed in sham controls (Anker et al., 2007; Larson and Carroll, 2007). Other studies have shown that estradiol replacement in ovariectomized rats either restores or enhances the motivation for cocaine, cocaine-conditioned place preference, and locomotor sensitization (for review, see Carroll and Anker, 2010; Carroll and Lynch, 2016; Harp et al., 2020). An obvious limitation of these studies is that ovariectomy depletes all ovarian hormones while chronic hormone replacement prevents the normal rhythmic cycling of ovarian hormones that normally occurs across the estrous cycle, making it difficult to compare these findings to those conducted in ovary-intact, naturally cycling female rats. As reviewed above, studies under the latter condition show that behavioral responding to cocaine is enhanced during estrus, a stage in which estrogen levels are declining from peak levels reached in proestrus, although the ratio of estradiol to progesterone is still slightly elevated (Broestl et al., 2018; Carney, 2019). To address this discrepancy, recent studies assessed the effects of chronic treatment with the estrogen receptor antagonist tamoxifen in ovary-intact, naturally cycling female rats on motivation to obtain cocaine and cue-induced reinstatement of cocaine-seeking behavior. Tamoxifen is an estrogen receptor modulator that antagonizes both estrogen receptors α and β and has been shown to inhibit estradiol-dependent behaviors such as mating behavior (Wilson et al., 2003). While chronic tamoxifen treatment prevented the increase in motivation for cocaine normally observed in female rats following cocaine self-administration, it did not reduce cocaine-seeking behavior during extinction and cue-induced reinstatement tests (Bakhti-Suroosh et al., 2019), in support of findings that other hormones such as progesterone may instead play a critical role (Feltenstein and See, 2007; Sinha et al., 2007). However, these studies involved chronic suppression of estradiol-dependent signaling, making it difficult to assess how changes in estradiol fluctuations across the estrous cycle influence relapse vulnerability. Studies assessing the effects of acute administration of estrogen receptor antagonists on relapse vulnerability in ovary-intact females are necessary to understand how transiently disrupting estrous cycle fluctuations influences relapse vulnerability.

### Underlying mechanisms of estrous cycle-dependent changes in relapse vulnerability

The cellular mechanisms that drive sex differences and estrous cycle-dependent changes in cocaine addiction liability and relapse vulnerability have been historically understudied, largely because of the exclusion of female subjects in preclinical research. In the past several years, there has been a shift toward systematically studying sex differences in preclinical addiction models. Recent studies have identified sex differences in mRNA and protein expression in stress systems and glutamatergic signaling pathways within the reward circuitry following cocaine exposure (Connelly and Unterwald, 2019, 2020; Castro-Zavala et al., 2020). Such studies will lay the foundation for future work focused on identifying cellular and synaptic mechanisms driving sex differences in behavioral responding to cocaine. The importance of such studies is further highlighted by recent evidence for latent sex differences in hippocampal synaptic plasticity despite common behavioral end points in males and females (Jain et al., 2019).

Dopaminergic projections from the ventral tegmental area (VTA) to the NAc are known to play a critical role in cocaine addiction, although less is known about the role dopamine plays in the incubation of cocaine craving (Carelli and West, 2014; Wolf, 2016). The basal firing rate of VTA dopamine (DA) neurons changes across the estrous cycle, with the greatest increase occurring in estrus, including an increase in burst firing (Zhang et al., 2008; Calipari et al., 2017), which are changes known to increase dopamine release (Pignatelli and Bonci, 2015). Similarly, DAergic transmission in the NAc changes across the estrous cycle (Becker and Hu, 2008). Importantly, cocaine-related cues can increase dopamine release in the NAc (Willuhn et al., 2010), and interactions between glutamatergic and dopaminergic signaling in the NAc are known to play a role in cocaine-seeking behavior (Anderson et al., 2008). Cocaine-induced dopamine release in the NAc has also been shown to be modulated by estrogenic signaling in the medial preoptic area of the hypothalamus in female rats (Tobiansky et al., 2016), indicating that neuroadaptations across the estrous cycle within this region could contribute to sex differences in incubated craving. While it is unknown how cue-induced DA release changes during withdrawal from extended access cocaine self-administration, it is likely that estrous cycle fluctuations during withdrawal impact dopaminergic transmission to influence cue-induced cocaine-seeking behavior.

Another brain area of interest is the basolateral amygdala (BLA), as it plays an important role in driving motivated behaviors like drug seeking. Human studies have shown that drug cues activate the amygdala (Childress et al., 1999) and that chronic cocaine use increases connectivity between the amygdala and limbic circuitry critical for driving-motivated behaviors like drug seeking (Konova et al., 2015; Contreras-Rodríguez et al., 2016). Projections from the BLA to the NAc have been shown to play an important role in incubated cue-induced cocaine craving in male rats (Lee et al., 2013; Ma et al., 2016) and other related studies have shown that inputs from the thalamus and cortex to the basal and/or lateral amygdala are critical in driving cue-induced reinstatement of drug seeking in male rats (Arguello et al., 2017; Rich et al., 2019, 2020). In addition, recent studies have shown an increase in BLA activity following early withdrawal (2–3 weeks) from extended-access cocaine self-administration in male rats, and that, when the magnitude of craving increases, so does BLA activity (Munshi et al., 2021). As with the VTA, BLA activity changes across the estrous cycle (Blume et al., 2017, 2019), raising the possibility of an interaction among cocaine exposure, BLA activity, and estrous cycle stage that may influence incubated craving. However, little is known regarding the cellular and synaptic mechanisms in the BLA contributing to time-dependent changes in cocaine craving in either males or females. Future studies will address these mechanisms in both sexes, including how they are influenced by hormonal fluctuations across the estrous cycle.

## Synthesis

Reviewing Editor: Karen Szumlinski, University of California at Santa Barbara Decisions are customarily a result of the Reviewing Editor and the peer reviewers coming together and discussing their recommendations until a consensus is reached. When revisions are invited, a fact-based synthesis statement explaining their decision and outlining what is needed to prepare a revision will be listed below. The following reviewer(s) agreed to reveal their identity: Yavin Shaham, Tod E Kippin.

Upon consultation with two reviewers with expertise in sex differences in cocaine-seeking/incubation of cocaine-seeking, we have reached the decision of Revise and Review. Both of the reviewers and I agree that the replication of sex differences in incubated cocaine-seeking under a 6 h-access paradigm is important and one of the major strengths of the report. The tracking of the estrous cycle is viewed as a strength that adds to the novelty of the report and all three of us agree that the report is very well-written, the statistics appropriate and the discussion of past and present findings concise, but comprehensive. Although the report had many strengths, both reviewers did raise some concerns that should be easily addressable within a 2 month time-frame. As highlighted below, Reviewer 1 points out some areas of improvement to the text that will augment clarity. Reviewer 2 expressed a more serious concern regarding the claim that the experimental design enabled the “tracking the emergence of sex differences", as there were no data points provided between the 1- and 15-day withdrawal time-point. This being said, we all acknowledge pandemic-related limitations on research and agree that a toning down of the claim that the “emergence” of sex differences is being tracked throughout the report and the re-branding of the report as a replication and extension of earlier work regarding sex differences in incubation would be wholly acceptable. Please find below the specific comments from both reviewers. We look forward to the revised manuscript.

Reviewer 1

This is an excellent paper in which the authors showed a critical role of the estrus phase in potentiation of incubation of craving in female rats. The authors extend previous work of Kerstetter et al. 2008 and Nicholas et al. 2019 on sex differences in incubation of craving and the role of estrous cycle in this effect. The paper has many strengths, including characterization the time course of the estrous cycle effect, a large n in the different experimental conditions, thorough assessment of the estrous cycle, the use of appropriate experimental design and proper statistical analyses, and a clear and scholarly description of the relevant literature. I could not find any experimental or statistical weaknesses in the study and enclose below several minor suggestions for the revision.

1. I suggest avoiding the use uncommon abbreviations like WD and instead spell them out. Uncommon abbreviations make it difficult for non-addiction researchers to follow the text.

2. Methods and other place in the text: please separate letters from numbers when reporting units (250g should be 250 g).

3: Methods, page 3: please report the number of rats that were excluded.

4. Results, page 8: I suggest changing ‘clearly indicate’ to ‘indicate’. The use of subjective words to describe the authors’ own results is distracting and unecessary. I also suggest changing ‘recovering addicts’ to ‘recovered users of drugs’ (the terminology used these days by human researchers and many preclinical researchers).

5. Discussion, page 9: I suggest revising the text in 2nd paragraph after considering the following. First, Kerstetter et al. 2008 used the original incubation procedure described in Grimm et al. 2001 (several hours of extinction responding without the cue and then 1 h of cue-induced reinstatement test; see also Lu et al. Neuropharmacology 2004 for other data using the original procedure). The second issue to consider is that as shown in Adhikary et al. 2017 (https://pubmed.ncbi.nlm.nih.gov/26989042/), operant extinction responding is the major driver of the incubated response under the experimental conditions of the authors, not the response to the cues. The data of Kerstetter et al. 2008 for extinction responding is similar to the authors’ current data and this should be emphasized instead of the differences with the cue-induced reinstatement test in the early study.

6. Discussion page 10: I suggest deleting “Together these findings indicate a clear role for estrogen in driving sex differences in cocaine addiction liability and relapse vulnerability.” The clinical evidence for this statement in reference to sex differences in human cocaine addiction is mixed at best. Many human studies on cue- or stress-induced craving in the lab or relapse (see studies of Rajita Sinha and others) do not support this statement. There is some evidence for menstrual cycle effect on craving and relapse in humans but even this evidence is mixed. And evidence for menstrual cycle effect on addiction-related behaviors does not necessary means that females are more vulnerable than males.

7. Figures: the authors should provide additional information in the figures to further describe their data: (1) Timecourse of infusions and active and inactive lever presses during training, (2) individual data for all bar graphs, and (3) time course of extinction responding during the incubation tests. Also, for all y-axes, please add session duration (operant response is a rate measure).

Reviewer 2

The present study examines the impact of withdrawal period following excessive intake on cocaine-seeking behavior in a rodent model of addiction. The study well-written, -designed, and the interpretations follow the findings. Overall, this is a very nice study. The study largely replicates prior findings with limited new information. However, the replication findings may be important enough on their own to have reasonable impact and should be of interest to researchers in the field.

For the most part, the study is well-designed but the study novelty is focused on tracking the emergence of sex/estrous cycle difference in incubated cocaine-seeking. To this end, there is inclusion of data for WD15 whereas a prior study of estrous modulation of incubated cocaine-seeking following “excessive” intake patterns first examined responding at WD29. However, there is no information about timepoints between WD1 and WD15 and incubated cocaine-seeking is known to emerge within the first week (Grimm et al., 2001) thus it is likely that the goal of tracking the emergence of sex differences in this behavior would necessitate further analyses.

The authors do a good job of summarizing the limited literature on sex differences and/or estrous modulation of incubated craving but there are appear to be a few small issues. First, several studies have examined WD1 responding in male and females, particularly those aimed at examination of reinstatement of cocaine-seeking. Thus, the higher responding on WD1 in females as a whole (when cycle is not assessed) or specifically in estrous has been reported several times. The authors do cite some of the papers but in passing and expanded description of the literature would strength the proposition that cocaine history has an important effect on suppressing estrous modulation of cocaine-seeking at the very earliest timepoint. Furthering this point, the authors do not discuss prior studies demonstrating estrous cycle perturbations produced by cocaine exposure which may explain the differences observed under limited versus excessive intake models.

The authors also highlight an important procedural consideration in the single study of incubation following limited intake versus the present: the use extinction without discrete cues prior to re-exposure to discrete cues versus initial (extinction) testing in the context and the discrete cues---which is accurate. However, they argue that “Here we systematically assessed withdrawal-dependent changes in cue-induced drug seeking behavior in response to the cocaine associated cue alone following extended-access self-administration and no extinction training” which appears incorrect or at least inaccurate as the present tests involved re-exposure to contextual and discrete cues during extinction; i.e. it is unclear what is meant by “alone”. This is a nuanced point but worth specifying since there maybe behavioral consequences-generally, initial testing (regardless of discrete cue presentation) appears to support time-dependent increases in cocaine-seeking.

Relatedly, they refer to their study being comparable to “standard incubation time-course” however, it is unclear what is meant by “standard” as experimental procedures vary across studies; for instance, Grimm et al., 2001 employed the extinction (in absence of discrete cue) followed by contingent re-exposure to discreet cues as well as having subjects live in the test cages, had daily moderate (3 h/day) access to cocaine, and lever press based operants; the Lu et al study did use the same incubation tests as employed here. Thus, I would caution arguing for there being a “standard” in the field. Rather, it is important to stress that despite differences in cocaine self-administration procedures, similar time-dependent intensification of responding is observed.

The self-administration procedures employed included a time-out period following each cocaine infusion. The authors provide response data but do not indicate the number of reinforcers obtained (or the mg/kg intake) for males relative to females or across subgroups tested at different time points. These data are needed to ensure intakes did not differ across WD test timepoints.
